# Targeting the TLR4 axis with microbiota-oriented interventions and innovations in diabetes therapy: a narrative review

**DOI:** 10.3389/fimmu.2025.1701504

**Published:** 2025-12-11

**Authors:** Christos G. Nikolaidis, Despoina Gyriki, Elisavet Stavropoulou, Eleni Karlafti, Triantafyllos Didangelos, Christina Tsigalou, Anastasia Thanopoulou

**Affiliations:** 1 Diabetes Center, 1st Propaedeutic Department of Internal Medicine, Medical School, ‘AHEPA‘ University General Hospital, Aristotle University of Thessaloniki, Thessaloniki, Macedonia, Greece; 2 2nd Department of Medicine, Hippokration Hospital, National and Kapodistrian University of Athens, Athens, Greece; 3 Hepatogastroenterology Unit, Academic Department of Internal Medicine, General Oncology Hospital of Kifissia “Agioi Anargyroi”, National and Kapodistrian University of Athens, Athens, Greece; 4 Master Program in “Food, Nutrition and Microbiome”, Laboratory of Hygiene and Environmental Protection, Department of Medicine, Democritus University of Thrace, Alexandroupolis, Greece; 5 Infectious Diseases Service, Department of Medicine, Lausanne University Hospital, University of Lausanne, Lausanne, Switzerland; 6 Laboratory of Hygiene and Environmental Protection, Department of Medicine, Democritus University of Thrace, Alexandroupolis, Greece

**Keywords:** gut microbiota, TLR4, fecal microbiota transplantation, celastrol, berberine, diabetes mellitus, inflammation, gut-on-chip

## Abstract

The gut microbiota–Toll-like receptor 4(TLR4)–nuclear factor kappa B(NF-κB) signaling is a key controller of low-grade chronic inflammation and insulin resistance in type 1 (T1DM) and type 2 diabetes mellitus (T2DM). While TLR4-mediated inflammation contributes to both T1DM and T2DM, the bulk of microbiota-targeted interventions have been studied in T2DM. The focus of the current review is on T2DM, with relevant parallels in T1DM noted where appropriate. Modulation of this pathway by dietary natural bioactive molecules, fecal microbiota transplantation (FMT), and technological innovations hold therapeutic promise for the reconstitution of metabolic and immune homeostasis. Agents like celastrol, berberine, paeoniflorin, and licorice extract exhibit anti-inflammatory and antidiabetic effects by TLR4/Myeloid differentiation primary response 88(MyD88)/NF-κB signaling inhibition. FMT enhanced β-cell function and insulin sensitivity with evidence of immune-metabolic modulation. New technologies, like ingestible biosensors and gut-on-chip platforms, allow real-time monitoring and precision modulating of the microbiota. Gastric bypass-induced microbial remodeling is linked to long-term glycemic benefit. Pharmacological, surgical, and technological manipulation of gut microbiota–immune interactions is a potential complementary strategy to diabetes. The future encompasses personalized microbiota-matching, controlled FMT regimens, and incorporation of digital therapeutics into microbiome-based precision medicine.

## 1 Introduction

The term diabetes mellitus is derived from the Greek word diabētēs, which originally meant to siphon or pass through (referring to excessive urination), and the Latin word “mellitus,” which means sweet-honeyed (1). Type 1 and Type 2 diabetes are the two primary forms of the disease (2). Type 5 diabetes mellitus (T5DM) or malnutrition-related diabetes is a distinct severe insulin-deficient type that is caused by chronic undernutrition early in life. It is characterized by pancreatic development and deranging insulin signaling without autoimmunity or primary insulin resistance (3). There is also gestational diabetes mellitus, which develops during pregnancy and requires careful management. Other types result from genetic flaws in β-cell function, insulin action, exocrine pancreas illnesses, endocrinopathies, infections, medications, genetic disorders, and rare immune-mediated diabetes (2). Each type is further categorized: for example, type 1 diabetes is either idiopathic or fulminant, and hybrid forms are slowly evolving autoimmune diabetes (LADA) and ketosis-prone type 2 diabetes (4). By 2045, there will be 700 million diabetic people, and the disease’s global economic impact will continue to rise (5).

T1DM is a severe, lifelong condition due to insufficient endogenous insulin secretion by the pancreatic β-cells, typically through autoimmune destruction. T1DM more often develops in children and adolescents but may occur at any time. Remarkably, its occurrence is rising, with the peak age of diagnosis shifting toward younger individuals (6). According to a systematic review that took place in 2020 and included 193 studies, there is a significant heterogeneity in incidence and prevalence of T1DM, with the incidence being 15 per 100,000 populations and prevalence being 9.5 per 10,000 people. In Mobasseri et al.’s review, the highest prevalence was reported in Europe (12.2 per 10,000 population), and the highest incidence was reported in America (20 per 100,000 population) (7).

In contrast to T1DM due to autoimmune destruction of pancreatic β-cells, type 2 diabetes mellitus (T2DM) has a distinct pathophysiological mechanism affecting not only the pancreas but the metabolic organs too. T2DM is primarily due to insulin resistance and β-cell dysfunction, while lifestyle and metabolic factors are the primary causes of disease etiology and progression (8).

T2DM is the most prevalent form of diabetes, while T1DM accounts for approximately 10-15% of all cases. However, the most widespread form among children below the age of 15 is T1DM, since over 500,000 children across the globe are estimated to be affected by this form (9). T2DM is a major global chronic disease with its prevalence having tripled in recent decades and an estimated 463 million cases in 2019. The disease’s economic burden has also increased dramatically, with direct healthcare costs reaching US$760 billion in 2019. Notably, approximately 80% of T2DM cases occur in low-and middle-income nations and it is necessary to curtail the global acceleration in order to improve population health and well-being (10).

While the treatment of T1DM relies on insulin therapy, the management of T2DM typically begins with lifestyle modifications and progresses to pharmacological treatment. Insulin is generally introduced in later stages, when hyperglycemia is no longer controlled by diet and oral antidiabetic agents. A patient-centered approach is recommended, and although metformin traditionally remains the first-line agent, newer therapeutic options—such as sodium-glucose cotransporter 2 inhibitors (SGLT2i) and glucagon-like peptide-1 receptor agonists (GLP-1 RAs)—have emerged. These agents may also be used as first-line treatments, either alone or in combination, depending on patient comorbidities, thereby reshaping the treatment landscape (11).

T2DM is a global healthcare issue that requires better management approaches. Current therapies focus on glucose control, but recent researches suggest that gut microbiota manipulation may be feasible. Current pharmaceuticals’ therapeutic outcomes may be influenced by their impact on the microbiota population. Understanding the relationship between microbiota, drugs, and diet may provide more efficient methods for managing and preventing T2DM (12).

Artificial Intelligence (AI) is revolutionizing diabetes care by creating data-driven predictive models and enabling continuous remote monitoring in patients, enhancing glycemic control through customized digital therapeutics and increased self-management. AI also enables clinical decision-making as well as resource optimization, and supports the transition from conventional methodologies to precise targeted care in diabetes (13).

The differential diagnostic criteria for common kinds of diabetes, include the key differences in etiology, clinical presentation, inheritance, prevalence, pathophysiological mechanisms, age of onset, clinical presentation, comorbidities, and treatment approaches among these three types (14).

This review aims to synthesize current understanding of targeting the gut microbiota– Toll-like receptor 4 (TLR4) axis in diabetes. We first establish the definition; subsequently critically examine microbiota-modulating interventions and mechanisms. With that goal in mind, we discuss how each natural product or technology impacts TLR4 signaling (directly or indirectly), and we indicate areas where there is incomplete knowledge and opportunities for future research.

## 2 Materials and methods

This narrative literature review integrates evidence from selected studies identified through searches in PubMed, Scopus, and Google Scholar, for studies published up to September 2025. The following search terms were used alone or in combination for the literature review: “diabetes mellitus”, “type 2 diabetes”, “TLR4” “gut microbiota”, “fecal microbiota transplantation”, “gut-on-chip”, “*in vitro*”, “animal model”, “clinical trial”, “inflammation”, and “cytokines” (e.g., TNF-α, IL-6). Articles were selected on the basis of relevance to the topic of gut microbiota–TLR4 signaling in diabetes. Boolean operators (AND, OR) were utilized to refine the search outcomes. Exclusion criteria encompassed articles not in English, case reports, commentaries and editorials.

### 2.1 Physiological role of gut microbiota in metabolism and immunity

The term microbiota originates from the Greek words “mikros” (small) and “bios”(life) (15). Gut microbiota refers to the community of microorganisms that inhabit the gastrointestinal tract—primarily bacteria, but also parasites, fungi, archaea and, according to some interpretations, viruses (16). The term microbiome, on the other hand, generally encompasses not only the microbiota itself but also the collective genomes of these microorganisms, including viral components and, in some interpretations, their metabolic pathways. However no official consensus exists, and multiple interpretations of these terms are found in the literature with subtle variations. In this text we will primarily use the term gut microbiota mainly to its bacterial component, the so called bacteriome.

The gut microbiota is the most densely populated and extensively studied microbial community in the human body, comprising several trillion microorganisms (17). It varies throughout the gastrointestinal tract, with the colon having the highest number of microorganisms. These microorganisms interact with the immune system and play a role in digestion, metabolism, and immune function (18).

Although it is difficult to define a healthy gut microbiota, it is typically characterized by high taxonomic diversity, a rich microbial gene pool, and stability (16). Each individual has a unique gut microbiota that is shaped in early life by factors such as gestational age at birth, type of milk feeding, weaning periods, lifestyle choices, and other dietary habits. Numerous additional factors influence the microbiota throughout the lifespan (19). Individual differences arise from various factors, including dietary preferences, lifestyle, culture, gut types, body mass index (BMI), and level of physical activity (20). In breastfed infants, the microbiota is dominated by *Lactobacillus* and *Bifidobacterium* (a genus within Actinobacteria), which thrive on human milk oligosaccharides. Formula-fed infants exhibit a more diverse microbiota with increased Firmicutes, linked to differences in nutrient composition, and significantly lower concentrations of those bacteria in breastfed infants but higher concentrations of *Bacteroides* and Enterobacteriaceae (21). Transitioning to solid foods during toddlerhood introduces new dietary substrates, leading to a shift where Firmicutes (e.g., *Clostridium, Eubacterium*) and Bacteroidetes (e.g., *Prevotella, Porphyromonas*) become the predominant phyla, reflecting greater microbial diversity. Healthy adults maintain a balanced microbiota dominated by Firmicutes and Bacteroidetes, supporting metabolic and immune functions. In malnourished individuals, the microbiota shows a decline in beneficial bacteria (e.g., *Bifidobacterium*) and an increase in Proteobacteria, a marker of dysbiosis. Aging alters the microbiota further; elderly individuals (65–80 years) exhibit reduced microbial diversity, with a decrease in *Bifidobacterium* and an increase in Proteobacteria.

Overall, the phyla Firmicutes and Bacteroidetes constitute the majority of the healthy gut microbiota, followed by Verrucomicrobia and Actinobacteria. Although the populations of *Bifidobacterium*, Firmicutes, and *Fecalibacterium prausnitzii* tend to decline with aging—and levels of *Escherichia coli*, other Proteobacteria, and *Staphylococcus* species increase—the overall composition of the gut microbiota remains relatively stable from the third to the seventh decade of life. Obesity is associated with a higher Firmicutes-to-Bacteroidetes ratio, potentially linked to increased energy harvest (22). Longitudinal analysis showed a bidirectional association of gut microbiota composition with obesity. Baseline BMI was prospectively associated with the relative abundance of ten separate microbial species. Of these, there were three (*Lachnospiraceae bacterium* 3 1 57FAA CT1*, Clostridium hathewayi*, and *Megamonas* unclassified) that were associated with markers of insulin resistance. *Lachnospiraceae bacterium* 3 1 57FAA CT1 was negatively correlated with Homeostasis Model Assessment of Insulin Resistance (HOMA-IR) and fasting insulin, indicating it may be a mediator of the association between adiposity and insulin resistance and a drug target (23).

The microbiota performs several crucial functions essential for maintenance of human health. It provides protection against pathogens by strengthening the intestinal barrier (barrier effect), since many of its components compete for adhesion and nutrients, secrete bacteriocins, and can lower the intestinal pH (24). Metabolically, some of the bacterial components of the microbiota contribute to carbohydrate digestion, since some bacteria produce short-chain fatty acids, and synthesize essential vitamins such as B1, B6, B9, B12, and K. The microbiota also plays a role in regulating endocrine functions and bone density, as well as modulating and secreting neurotransmitters like gamma-aminobutyric acid (GABA), serotonin, and acetylcholine (25). Interestingly, many microorganisms possess cytochromes, indicating complex metabolic capacities (24, 26). Furthermore, the microbiota is vital for the maturation and training of the immune system by continuously stimulating immune responses, thereby shaping immune function.

Specifically, the gut-associated lymphoid tissue (GALT), located throughout the gastrointestinal tract, continuously interacts with and is triggered by the gut microbiota leading to multiple immunological pathways; when a healthy gut microbiota is maintained, this interaction promotes immunological pathways that preserve a balance between immune-regulating and pro-inflammatory responses. In contrast, dysbiosis (an imbalance in the microbial community) can disrupt this balance and contribute to inappropriate or chronic inflammation (16, 27), leading to liver condition diseases, colorectal cancer, as well as metabolic disorders (28).

**Figure 1:**
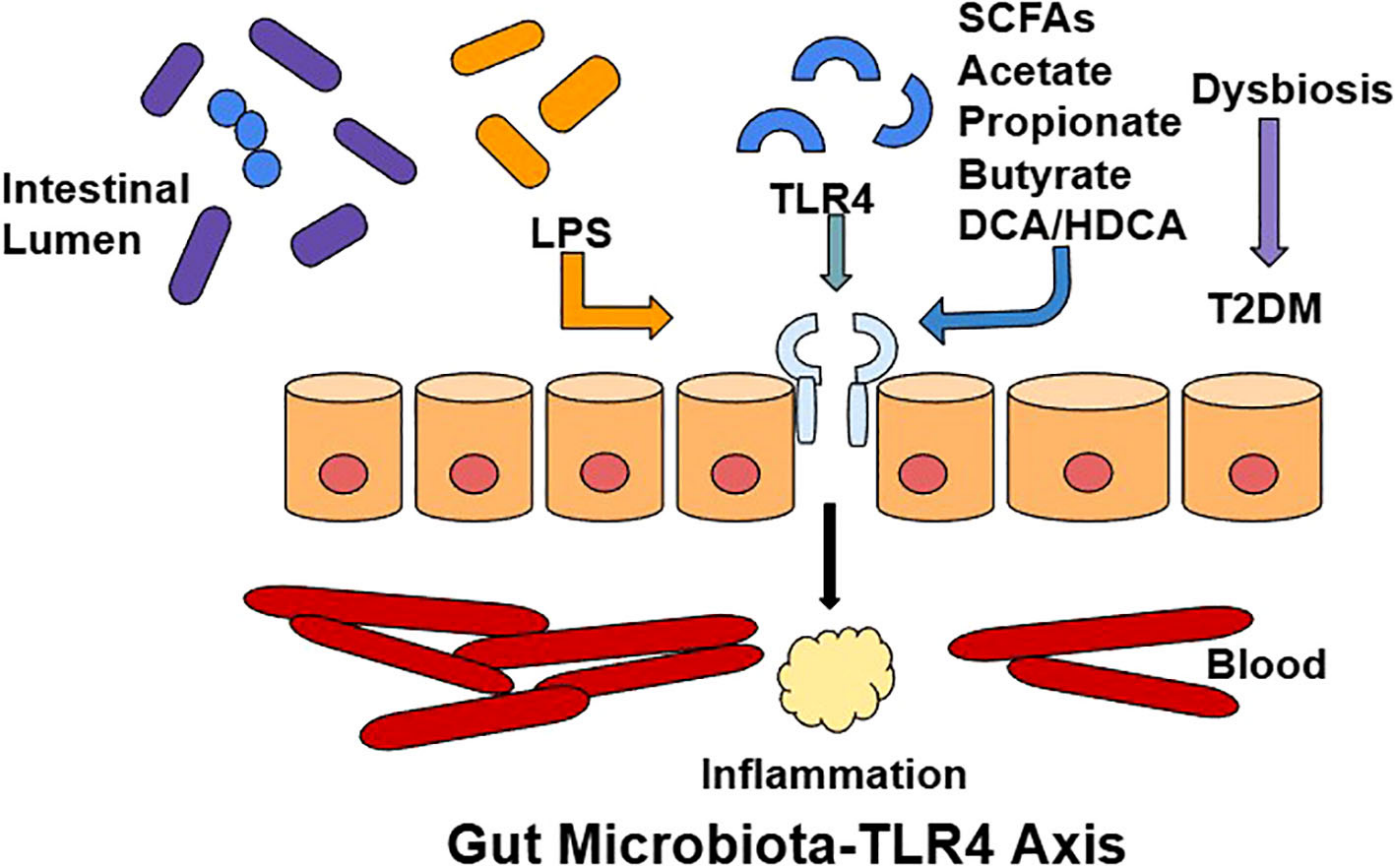
Gut Microbiota-TLR4/MyD88/NF-κB Axis: When gut dysbiosis occurs in the intestinal lumen, intestinal permeability is increased and there is an imbalance favoring lipopolysaccharide (LPS)-producing gram negative bacteria over short chain fatty acids (SFCA)-producing bacteria. LPS translocate into the blood circulation, activating TLR4 on immune and metabolic cells, which signals through the MyD88/NF-κB pathway, inducing pro-inflammatory cytokines production and chronic low-grade inflammation, thus impairing insulin signaling, promoting β-cell dysfunction and driving the development of diabetes. Additionally, the reduction of SFCA which help maintain barrier integrity and have anti-inflammatory properties, further exacerbates this immune and metabolic dysregulation.

A schematic representation of the gut microbiota–TLR4–NF-κB axis and its contribution to inflammation and insulin resistance is illustrated in **Figure 1**.

### 2.2 Targeting the gut microbiota-TLR4 axis in diabetes: natural products and herbal formulations as novel therapeutic approaches

Toll, a fly receptor, was first discovered in embryonic development. Toll-like receptors (TLRs), mammalian homologues, recognize microbial components, host defense against pathogens, and regulate sterile inflammation. Recognizing endogenous mediators is crucial for their function (29). Metabolic illnesses like obesity and T2DM often result in a persistent low-grade inflammatory state due to the activation of TLRs by Gram-negative bacteria’s lipopolysaccharides. This leads to increased reactive oxygen species and cytokine signaling, exacerbated by a diet high in fat, which lowers *Bifidobacterium* levels and increases inflammation (30). TLRs trigger both pro-diabetogenic and anti-diabetogenic signaling in commensal microbes and therefore regulate the microbiota composition and function and induce tolerance to self-antigens. The characterization of the precise molecular mechanisms by which TLR-mediated microbial interactions influence autoimmune processes can provide an entry point to targeted therapeutic approaches in autoimmune disease prevention, such as diabetes (31).

The gut microbiota uses TLR signaling to intensify metabolic inflammation when faced with a diet high in saturated fats (32). Three mechanisms initiate obesity-related metabolic inflammation: endoplasmic reticulum stress, TLR4 activation, and gut microbiota changes. TLR4 is central to the connection between dietary fat consumption, metabolic inflammation, and insulin resistance. Gut microbiota change can lead to compromised intestinal barrier function, and increased leakage of lipopolysaccharides and fatty acids, which activate systemic inflammation. Fatty acids can also initiate endoplasmic reticulum stress, which activates TLR4 (33). TLR4 is an extracellular receptor found in pancreatic islets, the brain, the liver, skeletal muscle, and adipose tissues. It regulates insulin sensitivity in these tissues, but its activation may inhibit insulin action by inducing pro-inflammatory mediators and Reactive Oxygen Species(ROS), which activate innate immune responses and insulin resistance development (34). Activation of TLR4 triggers mitogen-activated protein kinase(MAPK) pathways (Extracellular signal-regulated kinases (ERK)1/2, c-Jun N-terminal kinases(JNK), p38) that modulate insulin signaling, while evidence shows that deficiency of TLR4 prevents high-fat diet mice from metabolic endotoxemia, NF-κB activation, and insulin resistance (35).

A study examined the role of TLR pathways and gut microbiota in diabetes development in RIP-B7.1 transgenic mice. Results showed that mice lacking TLR3 and myeloid differentiation factor 88 (MyD88) were immune to diabetes, while TLR9-deficient mice showed more bacterial diversity and unique gut microbiomes (36). MyD88, a key protein in TLR signal transduction, activates inflammatory pathways, leading to higher diabetes risk in MyD88-knockout mice. High-fat diets increase insulin and cholesterol levels, leading to liver dysfunction. NF-κB, a key inflammatory marker, is also involved in inflammatory signaling. High TLR4 and NF-κB expression in T2DM rats lead to heart and liver complications. This suggests TLR4/NF-κB signaling may be related to T2DM (37).

The meta-analysis and systematic review of five RCTs found that traditional Chinese medicine (TCM) treatment in adults with T2DM enhanced significantly glycemic control (HbA1c, reductions in fasting and postprandial glucose levels), insulin resistance indices (HOMA-IR, HOMA-β), and gut microbiota modulation—most notably, an increase in *Bacteroides* abundance (38). TCM can exert hypoglycemic functions in part by regulating the structure of gut microbiota in a manner that suppresses metabolic endotoxemia, maintains intestinal mucosal barrier integrity, reduces Trimethylamine N-oxide (TMAO) and regulates bile acid metabolism (39). TCMs can also enhance T2DM by remodeling gut microbiota (increasing Short-chain fatty acids (SCFAs)-producing probiotics like *Akkermansia muciniphila*, preventing pathogens, enhancing intestinal barrier function, insulin sensitivity, lipid metabolism, and anti-inflammatory signaling) and their active saponins, flavonoids, polysaccharides, and alkaloids are biotransformed by microbes to amplify bioavailability and efficacy (40). However, with the paucity of few heterogeneous studies and sparse data on inflammatory markers and long-term safety, larger, well-designed trials are needed to reproduce these microbiota-mediated effects of TCM (38).

The gut microbiota degrades dietary fibers to SCFAs (acetate, propionate, and butyrate), which serve as energy substrates for colonic epithelium, regulate hormone secretion, and enhance barrier function (41, 42). SCFAs also bind G-protein-coupled receptors Free fatty acid receptor 3 (FFAR3, also termed GPR41) and G-protein coupled receptor 43 (GPR43) on host cells to enhance glucose uptake and stimulate the secretion of increretins (41, 43). However, dysbiosis associated with high-fat feeding primarily reduces SCFAs and potentially promotes gut leakage and endotoxemia (41, 44, 45). In addition, the microbiota metabolizes bile acids: microbial deconjugation and 7α-dehydroxylation produce secondary bile acids (like Hyodeoxycholic acid (HDCA) and deoxycholic acid) that signal through farnesoid X receptor (FXR) and TGR5 to regulate glucose homeostasis. Importantly, the discovery that HDCA binds and antagonizes TLR4 highlights a direct microbial–TLR4 crosstalk. Nonetheless, the gut microbes greatly regulate systemic metabolism and immune functions through the action of SCFAs, bile acids, and endotoxins (LPS) (41, 46–49).

Decreased SCFA producers (like *Roseburia* and *Faecalibacterium*) and increased Proteobacteria are associated with obesity and T2DM (41, 50, 51). Also, increased gut permeability (“leaky gut”) allows LPS to enter the circulation and activate TLR4 on macrophages and adipocytes leading to chronic inflammation (51). This has been studied in animals and humans, where the infusion of LPS increased insulin resistance and stopping the action of TLR4 reversed these actions. Therefore, restoring a healthy microbiota and strengthening the gut barrier may reduce LPS-mediated TLR4 activation, potentially breaking a key inflammatory loop in diabetes (52, 53).

Celastrol(C29H38O4) emerged as a potential therapeutic agent to regulate diabetic liver damage, and the TLR4/MyD88/NF-κB signaling pathway is identified as a novel putative target. Celastrol prevented proinflammatory growth in hepatic tissue, hence protecting the rats with T2DM from harm to their target organs (54). Celastrol, a triterpene from Tripterygium wilfordii Hook F [a traditional Chinese herbal medicine used as an immunosuppressive agent (55)], acts on cellular signaling for anti-inflammatory, anti-insulin resistance, and cardiometabolic effects. Faheem et al. proved that in streptozotocin-induced diabetic rats, celastrol strongly protected against testicular damage by suppressing TLR4/MyD88/NF-κB-mediated inflammation, oxidative stress, and apoptosis, and thereby restored metabolic, hormonal, and histopathological integrity (56).

Berberine, a natural product extracted from traditional Chinese herbs, was reported to enhance fasting blood glucose, triglyceride, and LDL cholesterol levels and insulin resistance in obese rats subjected to a high-fat diet through modulation of gut microbiota and inhibition of the LPS/TLR4/Tumor necrosis factor (TNF)-α pathway in the liver. Though it failed to exhibit remarkable alteration of body weight and visceral fat accumulation, berberine contributed to the alleviation of hepatic steatosis through the regulation of gut microbes and inhibition of inflammation, and this implies its therapeutic effect in alleviating insulin resistance by regulation of the gut-liver axis (57). In pre-diabetic Zucker Diabetic Fatty rats, a three-week berberine treatment markedly delayed the onset of overt diabetes, decreased food intake, fasting glucose, insulin resistance, and circulating LPS, and enhanced basal and glutamine-stimulated GLP-2 secretion. Mechanistically, berberine preserved intestinal barrier integrity (by enhancing goblet cells, villus length, mucin, ZO-1, and occluding) and decreased inflammatory signaling (TLR-4, NF-κB, TNF-α), in parallel with a restoration of gut microbiota composition and diversity (58). These outcomes related to microbiota and TLR4 signaling together make berberine a prototypic natural modulator of TLR4.

Licorice, which is derived from roots and rhizomes of plants belonging to the genus Glycyrrhiza, is a popular herbal medicine and sweetener. Licorice extract showed a dose-dependent hypoglycemic effect in type 2 diabetic mice with enhanced fasting glucose, insulin resistance, and lipid profiles, along with reduced colonic inflammation. These therapeutic properties have been found to be related to gut microbiota modulation and inhibition of the colonic TLR4/NF-κB pathway, hinting towards licorice’s potential use as a food additive in managing T2DM (59). Therefore, part of licorice’s hypoglycemic benefit can be attributed to attenuating LPS-TLR4 inflammatory processes in the gut.

A purified polysaccharide fraction from Cordyceps militaris (acidic-extractable polysaccharides (AEPSa)-a nourishing herb in traditional Chinese medicine (TCM) (60)- had hypoglycemic effects in T2DM mice via modulating the gut microbiota composition, enhancing the expression of tight junction proteins, and inhibiting colonic TLR4/NF-κB signaling. The findings, supported by FMT, suggest AEPSa as a putative prebiotic to improve glucose and lipid metabolism as well as intestinal barrier function in T2DM (61).

As a conventional Chinese medicine, sea cucumbers and sea cucumber extracts, such as polysaccharides and saponins, were reported to be recently endowed with anti-cancer, anti-inflammatory, and anti-oxidant activities (62). Holothuria leucospilota polysaccharide is a tropical edible sea cucumber species that showed antidiabetic activities by lowering blood glucose, increasing serum markers, and reversing hyperglycemia-induced tissue damage. These activities appear to be mediated through modulation of the Peroxisome Proliferator-Activated Receptors (PPARs)/(phosphoinositide-3 kinase(PI3K)/AKT pathway and gut microbiota, such as enhanced SCFA production and reduced pathogenic bacteria (63).

Sanziguben polysaccharides (SZP), derived from a traditional Chinese prescription for diabetic nephropathy (DN), exhibit protective effects by promoting gut microbiota balance and intestinal barrier integrity. These benefits likely stem from reduced LPS levels and inhibition of the TLR4/NF-κB/NOD-like Receptor Pyrin Domain-containing protein 3 (NLRP3) signaling pathway, suggesting SZP’s potential as a natural therapeutic option for DN (64). This indicates that SZP acts both by reducing microbial endotoxin input and by inhibiting downstream TLR4/NF-κB inflammatory signaling, underscoring its dual mechanisms of action along the axis.

Lycii fructus, or goji berry, or wolfberry, has been utilized in liver and kidney tonics, vision enhancement, and immunity improvement for centuries in traditional Chinese medicine. The major bioactive constituents in Lycii fructus are Lycium barbarum polysaccharides (LBP) (65). LBP cause dendritic cell maturation by upregulating Cluster of differentiation 80(CD80), Cluster of differentiation 86(CD86), and Major Histocompatibility Complex class II (MHC II) and enhancing TLR4, p38, Erk1/2, JNK, and Blimp1 expression, predominantly via the TLR4–Erk1/2–Blimp1 signaling pathway (66). LBPs showed antidiabetic effects in diet- and streptozotocin-induced diabetic mice by lowering fasting blood glucose, enhancing β-cell function, and augmenting gut beneficial bacteria, particularly *Allobaculum*. These alterations were correlated with increased intestinal barrier function via improved zonula occludens 1 expression, and FMT confirmed gut microbiota mediating glycemic modulation of LBPs (67).

Baihu Renshen Decoction (BHRS) is one of the oldest classic prescription of traditional Chinese medicine, which is used to treat diabetes (68). The research by Yao et al. illustrated that the BHRS was able to profoundly alleviate T2DM symptoms in rats by alleviating hyperglycemia, hyperlipidemia, insulin resistance, tissue injury, oxidative stress, and inflammation. BHRS’s therapeutic effects are likely to be mediated through the recovery of the gut barrier—indicated by the increase in tight junction protein and decrease in serum LPS—inhibition of the TLR4/NF-κB inflammatory pathway, and recovery of gut microbiota homeostasis through the regulation of both phylum and genus levels (69).

Paeonia lactiflora Pallas has been used as a TCM to treat pain, inflammation and immune disorder for more than 1000 years in China. Total glycoside of paeony (TGP) is a medicine extracted from dried root of Paeonia lactiflora Pallas. Paeoniflorin (Pae) is the main active ingredient of TGP (70). Paeoniflorin (PF) treatment, administered via intraperitoneal injection, can modulate gut microbiota and inhibit the TLR4-MyD88/Toll/IL-1R domain-containing adaptor protein inducing IFN-β (TRIF) signaling pathway to mitigate T1DM development in mice, with the consequent reduced diabetes incidence and decreased islet inflammatory infiltration. PF also improved intestinal barrier integrity, regulated immune cell populations toward increased Treg cells and decreased T helper 1(Th1)/T helper 17(Th17) ratios, and altered the structure of microbiota (effects that were abrogated by LPS, suggesting that TLR4-mediated mechanisms underlie its therapeutic effect) (71).

To sum up, TCM offers an integrated control of T2DM with enhancement of glycemic control and insulin sensitivity, normalization of gut microbiota to increase beneficial taxa (e.g., *Bacteroides, Akkermansia, Allobaculum*) and reduce endotoxin-producing pathogens, strengthening intestinal barrier function by upregulating tight junction proteins and mucin secretion, and inhibiting pro-inflammatory signaling through the TLR4/MyD88/NF−κB (and NLRP3) pathways in gut and liver. Active compounds such as celastrol, berberine, paeoniflorin, Lycium barbarum polysaccharides, and Cordyceps militaris polysaccharides are biotransformed by gut microbiota to make them more bioavailable and potent—protection from hepatic, renal, testicular, and islet tissue injury in animal models—and prescriptive medications such as Baihu Renshen Decoction and Sanziguben polysaccharides synergistically rebalance microbiota, downregulate endotoxemia, and reestablish metabolic and inflammatory homeostasis; however, larger, well−designed clinical trials are needed to establish these microbiota−mediated benefits and assess long−term safety profiles. Together, these findings report the therapeutic value of gut microbiota–TLR4 targeting for comprehensive control of diabetes (**Table 1**).

**Table 1:** Overview of selected pharmacological interventions targeting the gut microbiota and TLR4/NF-κB signaling pathway in diabetes, highlighting their sources, mechanisms of action, therapeutic effects.

Intervention	Source	Mechanism of action	Therapeutic effects	Reference
Celastrol	Tripterygium wilfordii triterpene	Inhibits TLR4/MyD88/NF-κB signaling in liver and testis	Protects against hepatic and testicular damage; anti-inflammatory; improves insulin sensitivity	(54–56)
Berberine	Alkaloid from Coptis, Berberis spp.	Modulates gut microbiota; inhibits LPS/TLR4/TNF-α pathway in liver	Lowers fasting glucose, triglycerides, LDL; delays diabetes onset; preserves gut barrier; reduces systemic inflammation	(57, 58)
Licorice extract	Glycyrrhiza spp. Roots	Alters microbiota composition; suppresses colonic TLR4/NF-κB	Dose-dependent hypoglycemic effect; improves lipid profile; reduces colonic inflammation	(59)
Acidic polysaccharides (AEPSa)	Cordyceps militaris	Enhances tight junction proteins; inhibits colonic TLR4/NF-κB	Improves glucose and lipid metabolism; strengthens intestinal barrier; acts as a prebiotic	(60, 61)
Sea cucumber polysaccharides	Holothuria leucospilota	Modulates PPARs/PI3K/AKT pathways; enriches SCFA-producing microbes	Lowers blood glucose; reverses hyperglycemia-induced tissue damage; antioxidant and anti-inflammatory	(62, 63)
Sanziguben polysaccharides (SZP)	Traditional prescription for diabetic nephropathy	Restores microbiota balance; inhibits TLR4/NF-κB/NLRP3 signaling	Protects kidney function; reduces LPS; strengthens gut barrier	(64)
Lycium barbarum polysaccharides (LBP)	Lycii fructus (goji berry)	Activates TLR4–Erk1/2–Blimp1 in dendritic cells; modulates gut microbiota	Lowers fasting glucose; enhances β-cell function; increases beneficial bacteria (Allobaculum); improves barrier integrity	(65–67)
Baihu Renshen Decoction (BHRS)	Classic TCM formula (Anemarrhena, Gypsum, Ginseng…)	Recovers tight junction expression; inhibits TLR4/NF-κB inflammatory pathway	Alleviates hyperglycemia, hyperlipidemia, insulin resistance, oxidative stress and inflammation in T2DM models	(68, 69)
Paeoniflorin (PF)	Glycoside from Paeonia lactiflora root	Suppresses TLR4-MyD88/TRIF signaling; reshapes gut microbiota; enhances Treg/Th1-Th17 balance	Reduces T1DM incidence; decreases islet inflammation; improves intestinal barrier; immunomodulatory	(70, 71)

### 2.3 Microbiota-oriented interventions and innovations in diabetes therapy

Probiotics, synbiotics and other dietary interventions represent important approaches to gut microbiota modulation but are not included in the scope of this review. The present review aimed to highlight emerging, less-established, less studied and more technologically innovative interventions—such as implantable devices, FMT, gut-on-chip systems, and digital microbiome tools—which are comparatively underrepresented in the literature.

**Figure 2:**
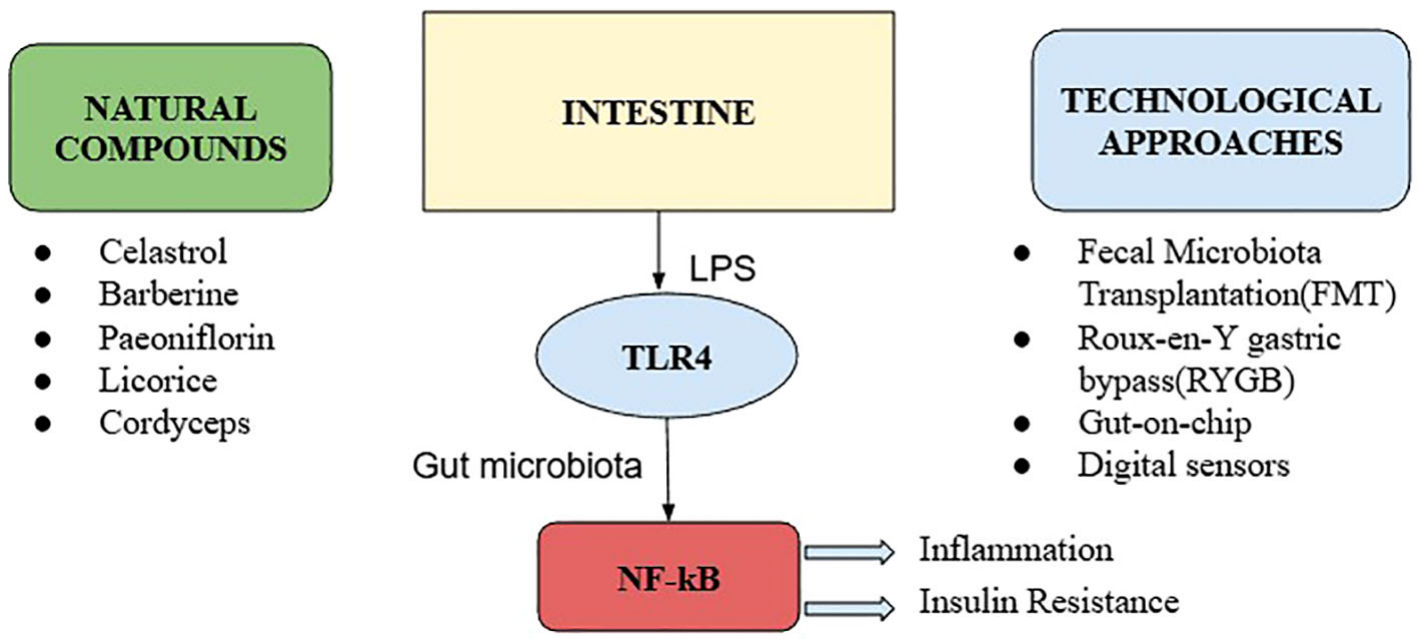
Integration of natural compounds with technological approaches.

**Figure 2** offers an integrated view of how natural bioactive compounds and technological innovations converge on the gut microbiota–TLR4 axis to modulate inflammation, metabolic signaling, and therapeutic outcomes in diabetes.

#### 2.3.1 Implanting electronic devices in the intestine for the treatment of T2DM

Although bariatric surgery remains the most effective and long-lasting therapy for obesity and T2DM, less invasive alternatives such as intragastric balloons, vagal blockade, and duodenal-jejunal bypass sleeves are being investigated but offer only temporary weight loss. Emerging endoscopic devices and therapies such as duodenal mucosal ablation will also have a role in managing obesity but likely with repeated applications or as one component of a multi-faceted treatment strategy to be effective (72).

Implantable electronic gastro-intestinal devices are a promising technology for T2DM management through targeted, real-time therapy by electrical stimulation of intestinal nerves and hormone regulation, but their invasive nature and need for periodic replacement limit extensive use. Future developments will focus on miniaturization, the integration of multiple stimulation methods, and feedback mechanisms to offer improved safety, precision, and patient acceptability (73).

Traditional fecal analysis can be inadequate in terms of predicting the dynamic environment of the GI tract, and thus there is interest in ingestible sensors for real-time microbiome monitoring (74). Chronic excess production of cortisol by way of long-standing stress is a key contributory factor in disorders like anxiety, Post-traumatic stress disorder(PTSD), metabolic syndrome, diabetes, immune suppression, and cardiovascular disease, so timely detection and treatment against the prolonged cortisol spikes may avoid these endocrine diseases. Using cortisol-sensitive genetic elements from *Clostridium scindens* in genetically modified *E. coli* Nissle 1917 probiotic—capable of detecting elevated cortisol and producing neuromodulators like tryptamine and serotonin—researchers created a cognitive, sense−and−respond microbe not only reporting cortisol levels by instant glow but also generating mood−enhancing metabolites to potentially restore hormonal homeostasis and modulate the gut–brain axis (75).

Microfluidics is being combined with 3D bioprinting and induced pluripotent stem cell (iPSC)-derived organoids to create even more physiologically relevant gut models. A recent study Human Microbial Cross-talk (HuMiX) was able to co-culture a full human microbiota with intestinal tissue, monitoring metabolite exchange (76). Gut-on-chip systems mimic diabetes-related alterations in the intestine, such as increased baseline permeability and augmented cytokine secretion (77, 78). Over the past decade, gut-on-chip (GOC) devices have developed more robustly in mimicking intestinal function but are still faced with challenges such as the determination of fully biocompatible materials, optimization of microfluidic design for true mechanical and chemical signaling, inclusion of real-time multiparametric sensors, and improvement of standardization and throughput (79). GOC platforms enable the dynamic, real-time study of human intestinal physiology and pathophysiology, including host-microbiome and immune interactions, under controlled mechanical and biochemical conditions, with the capacity to model disease states and test therapeutic interventions (80, 81). During diabetes conditions, chips exhibit compromised tight junctions and increased inflammatory signaling (77). Certain systems also incorporate real-time biosensors to dynamically track cytokines and microbial metabolites (79). These platforms enable precise evaluation of TLR4 and NF-κB signaling following exposure to lipopolysaccharide (LPS) or pathobionts, offering informative insight into mechanisms that maintain intestinal barrier function (82, 83). GOC technology can proceed more rapidly toward clinical application in the next few years, contributing to improved disease modeling, drug discovery, precision medicine, and reduced animal testing (79).

#### 2.3.2 FMT in metabolic syndrome and diabetes

Fecal microbiota transplantation (FMT) has a long history that goes back as far as the 4th century in China and at least the 17th century for use in veterinary medicine. Although FMT had already been suggested for treatment of pseudomembranous colitis by the surgeon Eisenman in 1958 (84), the first reported case of proven *Clostridioides difficile* infection treated with FMT occurred in 1983 (85). FMT has FDA-approved indication for this use since 2013 (86) and is also supported by scientific society guidelines (87, 88).

FMT is the procedure of transferring the entire gut microbiota of a healthy donor to a receiver (19). Microbiota transplantation may be administered via the upper gut, mid-gut, and lower gut. The most frequent method is oral administration of capsules, and the chosen microbiota may be powdered or in suspension. Endoscopy, nasojejunal tube, mid-gut transendoscopic enteral tubing (TET), small intestine stoma, or PEG-J may be utilized for infusions. Colonoscopy, enema, distal ileum stoma, colostomy, and colonic TET may be used to administer fecal microbiota to the lower gut (89).

FMT has since seen considerable expansion of use because of its perceived “natural” approach and low-tech, relatively low-cost method. There is growing interest in the treatment of dysbiosis-related conditions such as metabolic syndrome, obesity, food allergy, IBD, and IBS, with ongoing clinical trials investigating FMT for these indications (85). FMT is currently a second-line treatment for irritable bowel syndrome, hepatic steatosis, and hepatic encephalopathy, and may improve other gut dysbiosis-related disorders (90). It is also used in obesity, metabolic syndrome, severe multiple sclerosis, autism, multidrug-resistant organisms infections, and multiple organ dysfunction. FMT has also demonstrated beneficial effects on melanoma in clinical trials and animal models. There is no absolute contraindication of FMT from the current clinical evidence (86). Furthermore, FMT has shown potential in slowing the reduction of the endogenous insulin secretion in newly diagnosed T1DM patients with disease duration up to 12 months. The paper by Groot P et al. demonstrates that several microbiota-derived plasma metabolites- and specific bacterial strains like *D. piger, B. stercoris, Prevotella* spp, and *S. oralis* are associated with preserved beta cell function and may serve as therapeutic targets. Moreover, FMT was linked with improvements in the immune-related molecules as well as the small intestinal genes (91). FMT reshapes the gut microbiota, increasing the abundance of beneficial bacteria, including Bifidobacterium and butyrate producers that are inversely related to glycemic indices and directly associated with improved metabolic profiles in T2DM (92, 93).

The use of stool banks helps overcome barriers related to cost and availability while the advent of capsule FMT may further increase its use by offering greater convenience and reduced patient reluctance. The development of standardized protocols for donor screening, stool preparation, and delivery methods is anticipated (90).

However, to achieve significant progress in the field of metabolic disorders, it is essential to address several critical stages:

- Examining the use of FMT in patients with varying severities of insulin resistance to T2DM-especially since current evidence has been largely obtained from male subjects with metabolic syndrome-
- Assessing gender-specific effects,
- Establishing the most suitable method for donor selection based on clinical data,
- Defining the patient population in the context of the transplantation procedure, ensuring that the procedure is capable of eliciting a robust response and
- Identifying and characterizing the nature of that response.

Patients whose profiles match these criteria may stand to gain the most from FMT (94).

There is one investigation that sought to determine whether FMT could enhance the health status of patients with T2DM. A T2DM mouse model was established by feeding mice a high‐fat diet and administering an intraperitoneal injection of streptozotocin; subsequently, the gut microbiota was realigned using FMT. The study tracked fasting blood glucose, oral glucose tolerance, and HbA1c levels, which highlighted the hypoglycemic role of FMT. In addition, fasting insulin and glucose were measured along with HOMA‐IR (insulin resistance), HOMA‐IS (insulin sensitivity), and HOMA‐β (β-cell function). The outcomes demonstrated that FMT effectively ameliorated insulin sensitivity, increased β-cell function, and decreased both inflammation and the β-cell death rate. These findings imply that FMT might provide a therapeutic method for T2DM by enhancing islet insulin sensitivity and restoring islet integrity (86). There are various randomized controlled trials and meta-analyses that proved the same outcomes that FMT was able to show improvement of glycemic parameters, including a reduction in fasting plasma glucose, postprandial blood glucose, HbA1c, and HOMA-IR, along with triglycerides and cholesterol in patients with T2DM (92, 95–97). Multiple FMTs in obese patients with T2DM were demonstrated in a randomized controlled trial to increase the magnitude and duration of microbiota engraftment and, in combination with lifestyle change, caused more desirable alterations in the recipients’ microbiota and lipid levels and liver stiffness (19, 93). According to the current clinical data, FMT is safe and transiently improves insulin sensitivity and metabolic parameters in T2DM. However, long-term efficacy and ideal patient selection are under investigation (98, 99).

Despite its potential, FMT faces several limitations. FMT is not a safe and efficient treatment option for gut dysbiosis due to uncertainty about ecological factors, timeline of development, and causal contribution to disease. Our understanding of FMTs is poorly developed, making it difficult to rationalize timing and dosing regimens. Exposure to pathologic immune responses, exposure to infectious and non-infectious diseases, and antibiotic pre-treatment risks are also concerns (100).

Advances such as filtered and washed microbiota transplantation, bacterial consortia, and personalized interventions (combined with dietary interventions and microbiota-guided supplements) offer more tailored and safer avenues to augment immune function and treatment outcomes (101). However, the benefit–risk ratio must be carefully assessed for each specific indication.

#### 2.3.3 Microbiota changes after Roux-en-Y gastric bypass and their impact on diabetes

The gut microflora is altered dramatically after Roux-en-Y gastric bypass surgery (RYGB). Metagenomic sequence analyses reveal that Proteobacteria are up-regulated after RYGB, while Firmicutes and Bacteroidetes are reduced. Particular alterations in the microorganisms were connected with blood glucose, triglycerides, cholesterol, and BMI (102).

Within three months post-RYGB in morbidly obese patients, the gastrointestinal microbiota becomes more diverse, exhibits a variable composition, and shows a higher potential to tolerate oxygen, and to utilize macro- and micro-nutrients. These enhanced microbial features persist throughout the first year post-RYGB (103).

Additionally, one study confirmed that obesity and type 2 diabetes are correlated with a higher preoperative abundance of Bacteroidetes (including 12 species comprising *Phocaeicola dorei, Bacteroides fragilis*, and *Bacteroides caecimuris*) in the gut microbiota. This microbial profile was associated with diminished glucose tolerance and insulin sensitivity, suggesting a causal association between gut microbiota composition and the late-term metabolic complications following RYBG (104). These results demonstrate the significant impact of RYGB on the makeup of the gut microbiota and offer new avenues for diagnosis and treatment.

The general multitude of therapeutic strategies aimed at the gut microbiota to manage and potentially prevent diabetes mellitus. The **Table 2** highlights the therapeutic potential of FMT, dietary modification, and novel modalities. As may be noted, while some of these therapies are already proven, others remain experimental and require clinical validation.

**Table 2:** Extended therapeutic strategies targeting gut microbiota.

Intervention	Mechanism	Clinical evidence/notes	Reference
Natural Modulators of TLR4/NF-κB Axis	Act on gut-liver-barrier interface, reduce inflammation, restore insulin sensitivity	Bioactive agents (Celastrol, Berberine, Licorice, AEPSa, HLP) improve metabolic outcomes by modulating microbiota, suppressing inflammatory pathways, and protecting liver/testes/colon integrity.	(54–71)
Implantable Devices	Stimulate gut nerves; hormone regulation	Experimental, invasive but promising tech	(72–79)
FMT	Resets entire microbiome composition	Experimental; potential in obesity and T2DM	(84–101)
Roux-en-Y Gastric Bypass (RYGB)	Alters bile acid metabolism and gut flora drastically	Induces rapid microbial remodeling and improves glycemic control	(102–104)

## 3 Discussion

In the existing literature, several researchers elaborate extensively on the significance of the gut microbiota–TLR4 axis in the onset and progression of diabetes mellitus and especially type 2 diabetes (T2DM). There is growing evidence to indicate that the dysbiosis of gut microflora, an imbalance in microbial composition, results in compromised integrity in the intestinal barrier. This breakdown allows the transfer of LPS into the systemic circulation, activating TLR4 and beginning the cascade of pro-inflammatory signaling resulting in insulin resistance and metabolic inflammation (33).

This review highlights how the gut microbiota–TLR4 axis is a multifaceted therapeutic target for diabetes. The key takeaways are:

- Dysbiosis to Inflammation: The modulation of the gut microbiota–TLR4 axis attenuates chronic low-grade inflammation and engenders metabolic homeostasis. Interventions aimed at restoring microorganism balance (promoting SCFA producers, reducing endotoxin-producers) always reduce TLR4 activation.
- Natural Compounds: Natural products may act directly on pro-inflammatory pathways and are also transformed by intestinal microbes into metabolites even more active. For example, polysaccharides obtained from Dendrobium officinale have been shown to alleviate glucolipid metabolism disorder and decrease LPS leakage, thereby lessening metabolic inflammation in T2DM animal models (105). These products have a profound effect on reducing inflammation and oxidative stress, enhancing the junction proteins’ expression in the intestinal epithelial cells, and reducing intestinal permeability. As a result they improve insulin sensitivity and may reduce the autoimmune responses linked with diabetes (30).
- Digital and Mechanistic Innovation: The emerging electronic and microfluidic applications (e.g., gut-on-chip platforms and ingestible biosensors) have fostered the real-time monitoring and precision modulation of gut microbial activities, allowing for a new class of interventions known as “digital microbiome” that may complement existing pharmacotherapy.
- FMT and Diet: Dietary fiber treatment and microbiota transfer significantly reduce systemic LPS and inflammation, underlining the therapeutic potential of microbiome therapy. Bariatric surgery also alters gut microbiota to favor TLR4-suppressing metabolites.
- Clinical Targeting of TLR4: Despite enthusiasm, direct clinical targeting of TLR4 is untested. Recent human experiment of eritoran (a TLR4 antagonist) failed to improve insulin resistance (106), suggesting that simple blockade might be insufficient. This does not validate the axis, but highlights the complexity (e.g. duration, timing, or compensation). Combination strategies (e.g. TLR4 blockade plus microbiota modulation) might be required. There is ongoing research of more selective TLR4 inhibitors (lipid A analogs, small molecules) that, along with microbiota-directed therapies, could be investigated for application in metabolic disease.
- Immune Crosstalk: Crosstalk between innate immunity, TLR4, and metabolic regulation is intricate. As demonstrated, SCFAs and bile acids (like HDCA) are microbiota-produced signals that engage host receptors (GPRs, TGR5, FXR) and can modulate TLR4-mediated responses (41). This crosstalk means therapies could aim upstream (diet, fiber) or downstream (cytokine blockade) in this network.

In summary, targeting the gut microbiota-TLR4 axis is a fascinating potential complementary strategy for diabetes. By combining natural product pharmacology, microbial therapies, and contemporary biodesign, future opportunities will hopefully allow for personalized, durable, and mechanistic therapies that will move beyond the sole glucose-centric focus of contemporary therapies.

## 4 Limitations and future work

Although this narrative review followed no PRISMA-guided systematic protocol, nor did it include a formal approach to assess study quality or risk of bias, such as Cochrane ROB 2.0, it is possible that the review is limited by selection bias, a lack of reproducibility, and inconsistent quality by studies included in this review. Future work should resolve the methodological limitations that are outlined above.

Knowledge Gaps and Future Areas: There are still knowledge gaps to fill. It remains unclear which microbes or metabolites are most relevant to human TLR4 signaling, or which host factors modify these processes. Normalized microbiome analyses, biomarkers such as plasma LPS and levels of TLR4 expression would be essential in these aspects. Clinically, safety and efficacy data in patients treated with FMT and high-dose herbs would be valuable additions to our knowledge base. Crucially, there are no large randomized control trials to validate the efficacy of current microbiota-targeted therapies in human diseases.

These areas should form the subject matter of future studies:

- Personalized microbiome therapies: AI and deep sequencing can help detect specific microbiome signatures and personalize the therapy (prebiotics, probiotics, postbiotics, FMT) to the individual patient microbiome profile. “Microbiome consortia” can be designed for patients with unique TLR4-mediated inflammation phenotypes (107).
- Mechanistic biomarkers: Developing biomarkers of the TLR4 signaling pathway and the microbiome, such as circulating levels of MD-2 and the status of NF-κB, and SCFAs and bile acids, respectively.
- Digital and *in vitro* platforms: Broaden gut-on-chip technology to examine the interaction of multiple interventions, with the aim of identifying new small molecules to modulate the axis. For instance, gut-chip models with human cells and microbiota can be used to screen phytochemical compound libraries to identify new antagonists of TLR4.
- Prolonged clinical trials: Multicenter RCTs of promising therapeutic regimens (structured fiber supplements, next-generation synbiotics, TLR4 antagonists) with close follow-up of glycemic and immune parameters, together with evaluation of microbiome shifts. This would require standardized protocols (screening of donor for FMT, microorganism analyses, definition of endpoints) (108).

## 5 Conclusions and future directions

Finally, this review focuses on the pivotal role of gut microbiota dysbiosis in type 1 and type 2 diabetes pathogenesis, primarily based on chronic inflammatory-related mechanisms and intestinal barrier function impairment. Bioactive compounds of celastrol, berberine, paeoniflorin, and Lycium barbarum polysaccharides produce powerful anti-inflammatory and insulin-sensitizing effects through inhibition of TLR4/MyD88/NF-κB signaling, as well as restoration of gut barrier integrity and modulation of composition. FMT is also useful to even restore β cell function and insulin sensitivity, but considerations around donor selection, dosing and safety measures may limit its use. The intervention used in Roux-en-Y gastric bypass (RYGB) is effective in part due to long-lasting and important changes to gut microbiota, supporting the importance of gut microbial alterations as a central mechanism of action in therapeutic interventions that intervene with gut anatomy. As research continues to unveil the mechanisms by which the gut microbiome impacts glucose metabolism, insulin sensitivity, and systemic inflammation, the potential for microbiota-targeted therapies becomes increasingly evident. However, the variability of gut microbiota between individuals highlights the need for personalized therapeutic strategies tailored to the unique microbial and metabolic profiles of each patient.

Despite these insights, the current body of research is constrained by heterogeneity in study designs, limited sample sizes, and a paucity of long-term randomized controlled trials (RCTs), which collectively hinder the generalizability of findings. To advance the field, future research should prioritize multicenter RCTs of FMT in prediabetic individuals, development of standardized protocols for microbiome sequencing and metabolomic profiling, and examination of personalized synbiotic regimens according to individual gut microbiota compositions.

## References

[1] Polyzos SA Mantzoros CS . Diabetes mellitus: 100 years since the discovery of insulin. Metabolism. (2021) 118:154737. doi: 10.1016/j.metabol.2021.154737, PMID: 33610498

[2] American Diabetes Association . Diagnosis and classification of diabetes mellitus. Diabetes Care. (2013) 37:S81–90. doi: 10.2337/dc14-S081, PMID: 24357215

[3] Prajitno JH Sutanto H . Type 5 diabetes as a growing malnutrition driven health crisis in low and middle income countries. J Diabetes Metab Disord. (2025) 24:162. doi: 10.1007/s40200-025-01674-w, PMID: 40657327 PMC12246272

[4] Antar SA Ashour NA Sharaky M Khattab M Ashour NA Zaid RT . Diabetes mellitus: Classification, mediators, and complications; A gate to identify potential targets for the development of new effective treatments. Biomed Pharmacother. (2023) 168:115734. doi: 10.1016/j.biopha.2023.115734, PMID: 37857245

[5] Zhang L Chu J Hao W Zhang J Li H Yang C . Gut microbiota and type 2 diabetes mellitus: association, mechanism, and translational applications. Mediators Inflammation. (2021) 2021:5110276. doi: 10.1155/2021/5110276, PMID: 34447287 PMC8384524

[6] Ilonen J Lempainen J Veijola R . The heterogeneous pathogenesis of type 1 diabetes mellitus. Nat Rev Endocrinol. (2019) 15:635–50. doi: 10.1038/s41574-019-0254-y, PMID: 31534209

[7] Mobasseri M Shirmohammadi M Amiri T Vahed N Hosseini Fard H Ghojazadeh M . Prevalence and incidence of type 1 diabetes in the world: a systematic review and meta-analysis. Health Promot Perspect. (2020) 10:98–115. doi: 10.34172/hpp.2020.18, PMID: 32296622 PMC7146037

[8] Demir S Nawroth PP Herzig S Ekim Üstünel B . Emerging targets in type 2 diabetes and diabetic complications. Advanced Sci. (2021) 8:2100275. doi: 10.1002/advs.202100275, PMID: 34319011 PMC8456215

[9] Katsarou A Gudbjörnsdottir S Rawshani A Dabelea D Bonifacio E Anderson BJ . Type 1 diabetes mellitus. Nat Rev Dis Primers. (2017) 3:1–17. doi: 10.1038/nrdp.2017.16, PMID: 28358037

[10] Tinajero MG Malik VS . An update on the epidemiology of type 2 diabetes. Endocrinol Metab Clinics North America. (2021) 50:337–55. doi: 10.1016/j.ecl.2021.05.013, PMID: 34399949

[11] Davies MJ Aroda VR Collins BS Gabbay RA Green J Maruthur NM . Management of hyperglycemia in type 2 diabetes, 2022. A consensus report by the American diabetes association (ADA) and the European association for the study of diabetes (EASD). Diabetes Care. (2022) 45:2753–86. doi: 10.2337/dci22-0034, PMID: 36148880 PMC10008140

[12] Adeshirlarijaney A Gewirtz AT . Considering gut microbiota in treatment of type 2 diabetes mellitus. Gut Microbes. (2020) 11:253–64. doi: 10.1080/19490976.2020.1717719, PMID: 32005089 PMC7524291

[13] Ellahham S . Artificial intelligence: the future for diabetes care. Am J Med. (2020) 133:895–900. doi: 10.1016/j.amjmed.2020.03.033, PMID: 32325045

[14] Petersmann A Müller-Wieland D Müller UA Landgraf R Nauck M Freckmann G . Definition, classification and diagnosis of diabetes mellitus. Exp Clin Endocrinol Diabetes. (2019) 127:S1–7. doi: 10.1055/a-1018-9078, PMID: 31860923

[15] Barengolts E . Gut microbiota, prebiotics, probiotics, and synbiotics in management of obesity and prediabetes: review of randomized controlled trials. Endocr Pract. (2016) 22:1224–34. doi: 10.4158/EP151157.RA, PMID: 27409822

[16] Hou K Wu Z-X Chen X-Y Wang J-Q Zhang D Xiao C . Microbiota in health and diseases. Sig Transduct Target Ther. (2022) 7:135. doi: 10.1038/s41392-022-00974-4, PMID: 35461318 PMC9034083

[17] Lynch SV Pedersen O . The human intestinal microbiome in health and disease. New Engl J Med. (2016) 375:2369–79. doi: 10.1056/NEJMra1600266, PMID: 27974040

[18] Gyriki D Nikolaidis C Stavropoulou E Bezirtzoglou I Tsigalou C Vradelis S . Exploring the gut microbiome’s role in inflammatory bowel disease: insights and interventions. J Personalized Med. (2024) 14:507. doi: 10.3390/jpm14050507, PMID: 38793089 PMC11122163

[19] Gyriki D Nikolaidis CG Bezirtzoglou E Voidarou C Stavropoulou E Tsigalou C . The gut microbiota and aging: interactions, implications, and interventions. Front Aging. (2025) 6:1452917. doi: 10.3389/fragi.2025.1452917, PMID: 40438731 PMC12116569

[20] Rinninella E Raoul P Cintoni M Franceschi F Miggiano GAD Gasbarrini A . What is the healthy gut microbiota composition? A changing ecosystem across age, environment, diet, and diseases. Microorganisms. (2019) 7:14. doi: 10.3390/microorganisms7010014, PMID: 30634578 PMC6351938

[21] Gülden E Wong FS Wen L . The gut microbiota and Type 1 Diabetes. Clin Immunol. (2015) 159:143–53. doi: 10.1016/j.clim.2015.05.013, PMID: 26051037 PMC4761565

[22] Jandhyala SM Talukdar R Subramanyam C Vuyyuru H Sasikala M Reddy DN . Role of the normal gut microbiota. World J Gastroenterol. (2015) 21:8787–803. doi: 10.3748/wjg.v21.i29.8787, PMID: 26269668 PMC4528021

[23] Chen Z Radjabzadeh D Chen L Kurilshikov A Kavousi M Ahmadizar F . Association of insulin resistance and type 2 diabetes with gut microbial diversity: A microbiome-wide analysis from population studies. JAMA Netw Open. (2021) 4:e2118811. doi: 10.1001/jamanetworkopen.2021.18811, PMID: 34323983 PMC8322996

[24] Stavropoulou E Bezirtzoglou E . Probiotics in medicine: A long debate. Front Immunol. (2020) 11:2192. doi: 10.3389/fimmu.2020.02192, PMID: 33072084 PMC7544950

[25] Neuman H Debelius JW Knight R Koren O . Microbial endocrinology: the interplay between the microbiota and the endocrine system. FEMS Microbiol Rev. (2015) 39:509–21. doi: 10.1093/femsre/fuu010, PMID: 25701044

[26] Stavropoulou E Pircalabioru GG Bezirtzoglou E . The role of cytochromes P450 in infection. Front Immunol. (2018) 9:89. doi: 10.3389/fimmu.2018.00089, PMID: 29445375 PMC5797775

[27] Bemark M Pitcher MJ Dionisi C Spencer J . Gut-associated lymphoid tissue: a microbiota-driven hub of B cell immunity. Trends Immunol. (2024) 45:211–23. doi: 10.1016/j.it.2024.01.006, PMID: 38402045 PMC11227984

[28] Skoufou M Tsigalou C Vradelis S Bezirtzoglou E . The networked interaction between probiotics and intestine in health and disease: A promising success story. Microorganisms. (2024) 12:194. doi: 10.3390/microorganisms12010194, PMID: 38258020 PMC10818559

[29] Yu L Wang L Chen S . Endogenous toll-like receptor ligands and their biological significance. J Cell Mol Med. (2010) 14:2592–603. doi: 10.1111/j.1582-4934.2010.01127.x, PMID: 20629986 PMC4373479

[30] Gomes AC Bueno AA de Souza RGM Mota JF . Gut microbiota, probiotics and diabetes. Nutr J. (2014) 13:60. doi: 10.1186/1475-2891-13-60, PMID: 24939063 PMC4078018

[31] Burrows MP Volchkov P Kobayashi KS Chervonsky AV . Microbiota regulates type 1 diabetes through Toll-like receptors. Proc Natl Acad Sci USA. (2015) 112:9973–7. doi: 10.1073/pnas.1508740112, PMID: 26216961 PMC4538618

[32] Caesar R Tremaroli V Kovatcheva-Datchary P Cani PD Bäckhed F . Crosstalk between gut microbiota and dietary lipids aggravates WAT inflammation through TLR signaling. Cell Metab. (2015) 22:658–68. doi: 10.1016/j.cmet.2015.07.026, PMID: 26321659 PMC4598654

[33] Velloso LA Folli F Saad MJ . TLR4 at the crossroads of nutrients, gut microbiota, and metabolic inflammation. Endocr Rev. (2015) 36:245–71. doi: 10.1210/er.2014-1100, PMID: 25811237

[34] Rehman K Akash MSH . Mechanisms of inflammatory responses and development of insulin resistance: how are they interlinked? J BioMed Sci. (2016) 23:87. doi: 10.1186/s12929-016-0303-y, PMID: 27912756 PMC5135788

[35] Bezirtzoglou E Stavropoulou E Kantartzi K Tsigalou C Voidarou C Mitropoulou G . Maintaining digestive health in diabetes: the role of the gut microbiome and the challenge of functional foods. Microorganisms. (2021) 9:516. doi: 10.3390/microorganisms9030516, PMID: 33802371 PMC8001283

[36] Alkanani AK Hara N Lien E Ir D Kotter CV Robertson CE . Induction of diabetes in the RIP-B7.1 mouse model is critically dependent on TLR3 and myD88 pathways and is associated with alterations in the intestinal microbiome. Diabetes. (2014) 63:619–31. doi: 10.2337/db13-1007, PMID: 24353176

[37] Tian J Zhao Y Wang L Li L . Role of TLR4/MyD88/NF-κB signaling in heart and liver-related complications in a rat model of type 2 diabetes mellitus. J Int Med Res. (2021) 49:300060521997590. doi: 10.1177/0300060521997590, PMID: 33787393 PMC8020098

[38] Zheng Y Ding Q Wei Y Gou X Tian J Li M . Effect of traditional Chinese medicine on gut microbiota in adults with type 2 diabetes: A systematic review and meta-analysis. Phytomedicine. (2021) 88:153455. doi: 10.1016/j.phymed.2020.153455, PMID: 33478831

[39] Chen Y Wang M . New insights of anti-hyperglycemic agents and traditional Chinese medicine on gut microbiota in type 2 diabetes. Drug Des Devel Ther. (2021) 15:4849–63. doi: 10.2147/DDDT.S334325, PMID: 34876807 PMC8643148

[40] Su M Hu R Tang T Tang W Huang C . Review of the correlation between Chinese medicine and intestinal microbiota on the efficacy of diabetes mellitus. Front Endocrinol (Lausanne). (2023) 13:1085092. doi: 10.3389/fendo.2022.1085092, PMID: 36760813 PMC9905712

[41] Puddu A Sanguineti R Montecucco F Viviani GL . Evidence for the gut microbiota short-chain fatty acids as key pathophysiological molecules improving diabetes. Mediators Inflammation. (2014) 2014:162021. doi: 10.1155/2014/162021, PMID: 25214711 PMC4151858

[42] Ornelas A Countess JA Kim JY Colgan SP . Modifying microbially derived short chain fatty acids to promote health. J Physiol (2025). doi: 10.1113/JP287585, PMID: 40460326 PMC12797304

[43] Tolhurst G Heffron H Lam YS Parker HE Habib AM Diakogiannaki E . Short-chain fatty acids stimulate glucagon-like peptide-1 secretion via the G-protein–coupled receptor FFAR2. Diabetes. (2012) 61:364–71. doi: 10.2337/db11-1019, PMID: 22190648 PMC3266401

[44] Chae Y-R Lee YR Kim Y-S Park H-Y . Diet-induced gut dysbiosis and leaky gut syndrome. J Microbiol Biotechnol. (2024) 34:747–56. doi: 10.4014/jmb.2312.12031, PMID: 38321650 PMC11091682

[45] Gulhane M Murray L Lourie R Tong H Sheng YH Wang R . High fat diets induce colonic epithelial cell stress and inflammation that is reversed by IL-22. Sci Rep. (2016) 6:28990. doi: 10.1038/srep28990, PMID: 27350069 PMC4924095

[46] Chávez-Talavera O Tailleux A Lefebvre P Staels B . Bile acid control of metabolism and inflammation in obesity, type 2 diabetes, dyslipidemia and NAFLD Short title: Bile acids in meta-inflammatory disorders. Gastroenterology. (2017) 152:1679–1694.e3. doi: 10.1053/j.gastro.2017.01.055, PMID: 28214524

[47] Wahlström A Sayin SI Marschall H-U Bäckhed F . Intestinal crosstalk between bile acids and microbiota and its impact on host metabolism. Cell Metab. (2016) 24:41–50. doi: 10.1016/j.cmet.2016.05.005, PMID: 27320064

[48] Long SL Gahan CGM Joyce SA . Interactions between gut bacteria and bile in health and disease. Mol Aspects Med. (2017) 56:54–65. doi: 10.1016/j.mam.2017.06.002, PMID: 28602676

[49] Wise JL Cummings BP . The 7-α-dehydroxylation pathway: An integral component of gut bacterial bile acid metabolism and potential therapeutic target. Front Microbiol. (2023) 13:1093420. doi: 10.3389/fmicb.2022.1093420, PMID: 36699589 PMC9868651

[50] Gurung M Li Z You H Rodrigues R Jump DB Morgun A . Role of gut microbiota in type 2 diabetes pathophysiology. eBioMedicine. (2020) 51:102590. doi: 10.1016/j.ebiom.2019.11.051, PMID: 31901868 PMC6948163

[51] Mohammad S Thiemermann C . Role of metabolic endotoxemia in systemic inflammation and potential interventions. Front Immunol. (2021) 11:594150. doi: 10.3389/fimmu.2020.594150, PMID: 33505393 PMC7829348

[52] Raetzsch CF Brooks NL Alderman JM Moore KS Hosick PA Klebanov S . LPS inhibition of glucose production through the TLR4, MYD88, NFκB pathway. Hepatology. (2009) 50:592–600. doi: 10.1002/hep.22999, PMID: 19492426 PMC2822400

[53] Carvalho BM Guadagnini D Tsukumo DML Schenka AA Latuf-Filho P Vassallo J . Modulation of gut microbiota by antibiotics improves insulin signalling in high-fat fed mice. Diabetologia. (2012) 55:2823–34. doi: 10.1007/s00125-012-2648-4, PMID: 22828956

[54] Han L Li C Sun B Xie Y Guan Y Ma Z . Protective effects of celastrol on diabetic liver injury via TLR4/myD88/NF-κB signaling pathway in type 2 diabetic rats. J Diabetes Res. (2016) 2016:2641248. doi: 10.1155/2016/2641248, PMID: 27057550 PMC4745324

[55] Chen Y Gong Z Chen X Tang L Zhao X Yuan Q . Tripterygium wilfordii Hook F (a traditional Chinese medicine) for primary nephrotic syndrome. Cochrane Database Syst Rev (2013) CD008568. doi: 10.1002/14651858.CD008568.pub2/full, PMID: PMC13034419 23934958

[56] Faheem H Alawadhi R Basha EH Ismail R Ibrahim HA Elshamy AM . Ameliorating immune-dependent inflammation and apoptosis by targeting TLR4/MYD88/NF-κB pathway by celastrol mitigates the diabetic reproductive dysfunction. Physiol Genomics. (2025) 57:103–14. doi: 10.1152/physiolgenomics.00072.2024, PMID: 39510137

[57] Liu D Zhang Y Liu Y Hou L Li S Tian H . Berberine modulates gut microbiota and reduces insulin resistance via the TLR4 signaling pathway. Exp Clin Endocrinol Diabetes. (2018) 126:513–20. doi: 10.1055/s-0043-125066, PMID: 29365334

[58] Wang Y Liu H Zheng M Yang Y Ren H Kong Y . Berberine slows the progression of prediabetes to diabetes in zucker diabetic fatty rats by enhancing intestinal secretion of glucagon-like peptide-2 and improving the gut microbiota. Front Endocrinol (Lausanne). (2021) 12:609134. doi: 10.3389/fendo.2021.609134, PMID: 34025574 PMC8138858

[59] Zhang Y Xu Y Zhang L Chen Y Wu T Liu R . Licorice extract ameliorates hyperglycemia through reshaping gut microbiota structure and inhibiting TLR4/NF-κB signaling pathway in type 2 diabetic mice. Food Res Int. (2022) 153:110945. doi: 10.1016/j.foodres.2022.110945, PMID: 35227470

[60] Li Y He L Song H Bao X Niu S Bai J . Cordyceps: Alleviating ischemic cardiovascular and cerebrovascular injury - A comprehensive review. J Ethnopharmacol. (2024) 332:118321. doi: 10.1016/j.jep.2024.118321, PMID: 38735418

[61] Zhao H Li M Liu L Li D Zhao L Wu Z . Cordyceps militaris polysaccharide alleviates diabetic symptoms by regulating gut microbiota against TLR4/NF-κB pathway. Int J Biol Macromol. (2023) 230:123241. doi: 10.1016/j.ijbiomac.2023.123241, PMID: 36641024

[62] Ru R Guo Y Mao J Yu Z Huang W Cao X . Cancer cell inhibiting sea cucumber (Holothuria leucospilota) protein as a novel anti-cancer drug. Nutrients. (2022) 14:786. doi: 10.3390/nu14040786, PMID: 35215436 PMC8879703

[63] Zhao F Liu Q Cao J Xu Y Pei Z Fan H . A sea cucumber (Holothuria leucospilota) polysaccharide improves the gut microbiome to alleviate the symptoms of type 2 diabetes mellitus in Goto-Kakizaki rats. Food Chem Toxicol. (2020) 135:110886. doi: 10.1016/j.fct.2019.110886, PMID: 31626838

[64] Wang F Liu C Ren L Li Y Yang H Yu Y . Sanziguben polysaccharides improve diabetic nephropathy in mice by regulating gut microbiota to inhibit the TLR4/NF-κB/NLRP3 signalling pathway. Pharm Biol. (2023) 61:427–36. doi: 10.1080/13880209.2023.2174145, PMID: 36772833 PMC9930838

[65] Duan J-L Liu M-Q Liu Y-N Liang X-F Cao C Yao A-N . Comparative study on physicochemical characterization and immunomodulatory activities of neutral and acidic Lycium barbarum polysaccharides. Biomed Pharmacother. (2024) 181:117659. doi: 10.1016/j.biopha.2024.117659, PMID: 39486371

[66] Duan X Lan Y Zhang X Hou S Chen J Ma B . Lycium barbarum Polysaccharides Promote Maturity of Murine Dendritic Cells through Toll-Like Receptor 4-Erk1/2-Blimp1 Signaling Pathway. J Immunol Res. (2020) 2020:1751793. doi: 10.1155/2020/1751793, PMID: 33344654 PMC7725586

[67] Zhou W Yang T Xu W Huang Y Ran L Yan Y . The polysaccharides from the fruits of Lycium barbarum L. confer anti-diabetic effect by regulating gut microbiota and intestinal barrier. Carbohydr Polymers. (2022) 291:119626. doi: 10.1016/j.carbpol.2022.119626, PMID: 35698418

[68] Tian Y Zhong W Zhang Y Zhou L Fu X Wang L . Baihu Jia Renshen Decoction for type 2 diabetic mellitus. Med (Baltimore). (2020) 99:e20210. doi: 10.1097/MD.0000000000020210, PMID: 32384518 PMC7220408

[69] Yao B Pan B Tian T Su X Zhang S Li H . Baihu renshen decoction ameliorates type 2 diabetes mellitus in rats through affecting gut microbiota enhancing gut permeability and inhibiting TLR4/NF-κB-mediated inflammatory response. Front Cell Infect Microbiol. (2022) 12:1051962. doi: 10.3389/fcimb.2022.1051962, PMID: 36439213 PMC9691847

[70] Zhang L Wei W . Anti-inflammatory and immunoregulatory effects of paeoniflorin and total glucosides of paeony. Pharmacol Ther. (2020) 207:107452. doi: 10.1016/j.pharmthera.2019.107452, PMID: 31836457

[71] Luo C Yang D Hou C Tan T Chao C . Paeoniflorin protects NOD mice from T1D through regulating gut microbiota and TLR4 mediated myD88/TRIF pathway. Exp Cell Res. (2023) 422:113429. doi: 10.1016/j.yexcr.2022.113429, PMID: 36402426

[72] Brethauer SA Chang J Galvao Neto M Greve JW . Gastrointestinal devices for the treatment of type 2 diabetes. Surg Obes Relat Dis. (2016) 12:1256–61. doi: 10.1016/j.soard.2016.02.031, PMID: 27568475

[73] Xu T-C Liu Y Yu Z Xu B . Gut-targeted therapies for type 2 diabetes mellitus: A review. World J Clin cases. (2024) 12:1–8. doi: 10.12998/wjcc.v12.i1.1, PMID: 38292634 PMC10824172

[74] Bettinger CJ . Materials advances for next-generation ingestible electronic medical devices. Trends Biotechnol. (2015) 33:575–85. doi: 10.1016/j.tibtech.2015.07.008, PMID: 26403162

[75] Litteral V Migliozzi R Metzger D McPherson C Saldanha R . Engineering a cortisol sensing enteric probiotic. ACS Biomater Sci Eng. (2023) 9:5163–75. doi: 10.1021/acsbiomaterials.2c01300, PMID: 37647169

[76] Shah P Fritz JV Glaab E Desai MS Greenhalgh K Frachet A . A microfluidics-based *in vitro* model of the gastrointestinal human–microbe interface. Nat Commun. (2016) 7:11535. doi: 10.1038/ncomms11535, PMID: 27168102 PMC4865890

[77] Min S Than N Shin YC Hu G Shin W Ambrosini YM . Live probiotic bacteria administered in a pathomimetic Leaky Gut Chip ameliorate impaired epithelial barrier and mucosal inflammation. Sci Rep. (2022) 12:22641. doi: 10.1038/s41598-022-27300-w, PMID: 36587177 PMC9805460

[78] Hahn S Han IW Shin SH Kim G Kim JH . Modeling diabetic intestinal organoids: Aspects of rapid gut barrier disruption. Biochem Biophys Res Commun. (2025) 760:151730. doi: 10.1016/j.bbrc.2025.151730, PMID: 40168710

[79] Marrero D Pujol-Vila F Vera D Gabriel G Illa X Elizalde-Torrent A . Gut-on-a-chip: Mimicking and monitoring the human intestine. Biosens Bioelectronics. (2021) 181:113156. doi: 10.1016/j.bios.2021.113156, PMID: 33761417

[80] Wang L Wu J Chen J Dou W Zhao Q Han J . Advances in reconstructing intestinal functionalities *in vitro*: From two/three dimensional-cell culture platforms to human intestine-on-a-chip. Talanta. (2021) 226:122097. doi: 10.1016/j.talanta.2021.122097, PMID: 33676654

[81] Ashammakhi N Nasiri R Barros NR de Tebon P Thakor J Goudie M . Gut-on-a-chip: Current progress and future opportunities. Biomaterials. (2020) 255:120196. doi: 10.1016/j.biomaterials.2020.120196, PMID: 32623181 PMC7396314

[82] Kim HJ Li H Collins JJ Ingber DE . Contributions of microbiome and mechanical deformation to intestinal bacterial overgrowth and inflammation in a human gut-on-a-chip. Proc Natl Acad Sci U.S.A. (2016) 113:E7–E15. doi: 10.1073/pnas.1522193112, PMID: 26668389 PMC4711860

[83] De Gregorio V Sgambato C Urciuolo F Vecchione R Netti PA Imparato G . Immunoresponsive microbiota-gut-on-chip reproduces barrier dysfunction, stromal reshaping and probiotics translocation under inflammation. Biomaterials. (2022) 286:121573. doi: 10.1016/j.biomaterials.2022.121573, PMID: 35617781

[84] Eiseman B Silen W Bascom GS Kauvar AJ . Fecal enema as an adjunct in the treatment of pseudomembranous enterocolitis. Surgery. (1958) 44:854–9., PMID: 13592638

[85] Kelly CR Kahn S Kashyap P Laine L Rubin D Atreja A . Update on fecal microbiota transplantation 2015: indications, methodologies, mechanisms, and outlook. Gastroenterology. (2015) 149:223–37. doi: 10.1053/j.gastro.2015.05.008, PMID: 25982290 PMC4755303

[86] Wang H Lu Y Yan Y Tian S Zheng D Leng D . Promising treatment for type 2 diabetes: fecal microbiota transplantation reverses insulin resistance and impaired islets. Front Cell Infect Microbiol. (2020) 9:455. doi: 10.3389/fcimb.2019.00455, PMID: 32010641 PMC6979041

[87] van Prehn J Reigadas E Vogelzang EH Bouza E Hristea A Guery B . European Society of Clinical Microbiology and Infectious Diseases: 2021 update on the treatment guidance document for Clostridioides difficile infection in adults. Clin Microbiol Infect. (2021) 27:S1–S21. doi: 10.1016/j.cmi.2021.09.038, PMID: 34678515

[88] Johnson S Lavergne V Skinner AM Gonzales-Luna AJ Garey KW Kelly CP . Clinical practice guideline by the infectious diseases society of america (IDSA) and society for healthcare epidemiology of America (SHEA): 2021 focused update guidelines on management of clostridioides difficile infection in adults. Clin Infect Dis. (2021) 73:e1029–44. doi: 10.1093/cid/ciab549, PMID: 34164674

[89] Zhang F Cui B He X Nie Y Wu K Fan D . Microbiota transplantation: concept, methodology and strategy for its modernization. Protein Cell. (2018) 9:462–73. doi: 10.1007/s13238-018-0541-8, PMID: 29691757 PMC5960466

[90] Kim KO Gluck M . Fecal microbiota transplantation: an update on clinical practice. Clin Endosc. (2019) 52:137–43. doi: 10.5946/ce.2019.009, PMID: 30909689 PMC6453848

[91] /Groot P Nikolic T Pellegrini S Sordi V Imangaliyev S Rampanelli E . Faecal microbiota transplantation halts progression of human new-onset type 1 diabetes in a randomised controlled trial. Gut. (2021) 70:92–105. doi: 10.1136/gutjnl-2020-322630, PMID: 33106354 PMC7788262

[92] Su L Hong Z Zhou T Jian Y Xu M Zhang X . Health improvements of type 2 diabetic patients through diet and diet plus fecal microbiota transplantation. Sci Rep. (2022) 12:1152. doi: 10.1038/s41598-022-05127-9, PMID: 35064189 PMC8782834

[93] Ng SC Xu Z Mak JWY Yang K Liu Q Zuo T . Microbiota engraftment after faecal microbiota transplantation in obese subjects with type 2 diabetes: a 24-week, double-blind, randomised controlled trial. Gut. (2022) 71:716–23. doi: 10.1136/gutjnl-2020-323617, PMID: 33785557

[94] Aron-Wisnewsky J Clément K Nieuwdorp M . Fecal microbiota transplantation: a future therapeutic option for obesity/diabetes? Curr Diabetes Rep. (2019) 19:51. doi: 10.1007/s11892-019-1180-z, PMID: 31250122

[95] Wu Z Zhang B Chen F Xia R Zhu D Chen B . Fecal microbiota transplantation reverses insulin resistance in type 2 diabetes: A randomized, controlled, prospective study. Front Cell Infect Microbiol. (2023) 12:1089991. doi: 10.3389/fcimb.2022.1089991, PMID: 36704100 PMC9872724

[96] Ding D Yong H You N Lu W Yang X Ye X . Prospective study reveals host microbial determinants of clinical response to fecal microbiota transplant therapy in type 2 diabetes patients. Front Cell Infect Microbiol. (2022) 12:820367. doi: 10.3389/fcimb.2022.820367, PMID: 35402293 PMC8990819

[97] Zhang J Wang H Liu Y Shi M Zhang M Zhang H . Advances in fecal microbiota transplantation for the treatment of diabetes mellitus. Front Cell Infect Microbiol. (2024) 14:1370999. doi: 10.3389/fcimb.2024.1370999, PMID: 38660489 PMC11039806

[98] Qin L Fan B Zhou Y Zheng J Diao R Wang F . Targeted gut microbiome therapy: Applications and prospects of probiotics, fecal microbiota transplantation and natural products in the management of type 2 diabetes. Pharmacol Res. (2025) 213:107625. doi: 10.1016/j.phrs.2025.107625, PMID: 39875017

[99] Vassallo GA Dionisi T De Vita V Augello G Gasbarrini A Pitocco D . The role of fecal microbiota transplantation in diabetes. Acta Diabetol. (2025) 62:977–81. doi: 10.1007/s00592-025-02508-0, PMID: 40252102 PMC12283471

[100] Marrs T Walter J . Pros and cons: Is faecal microbiota transplantation a safe and efficient treatment option for gut dysbiosis? Allergy. (2021) 76:2312–7. doi: 10.1111/all.14750, PMID: 33483999

[101] Marroncini G Naldi L Martinelli S Amedei A . Gut–liver–pancreas axis crosstalk in health and disease: from the role of microbial metabolites to innovative microbiota manipulating strategies. Biomedicines. (2024) 12:1398. doi: 10.3390/biomedicines12071398, PMID: 39061972 PMC11273695

[102] Graessler J Qin Y Zhong H Zhang J Licinio J Wong M-L . Metagenomic sequencing of the human gut microbiome before and after bariatric surgery in obese patients with type 2 diabetes: correlation with inflammatory and metabolic parameters. Pharmacogenomics J. (2013) 13:514–22. doi: 10.1038/tpj.2012.43, PMID: 23032991

[103] Palleja A Kashani A Allin KH Nielsen T Zhang C Li Y . Roux-en-Y gastric bypass surgery of morbidly obese patients induces swift and persistent changes of the individual gut microbiota. Genome Med. (2016) 8:67. doi: 10.1186/s13073-016-0312-1, PMID: 27306058 PMC4908688

[104] Debédat J Le Roy T Voland L Belda E Alili R Adriouch S . The human gut microbiota contributes to type-2 diabetes non-resolution 5-years after Roux-en-Y gastric bypass. Gut Microbes. (2022) 14:2050635. doi: 10.1080/19490976.2022.2050635, PMID: 35435140 PMC9037437

[105] Chen X Chen C Fu X . Dendrobium officinale Polysaccharide Alleviates Type 2 Diabetes Mellitus by Restoring Gut Microbiota and Repairing Intestinal Barrier via the LPS/TLR4/TRIF/NF-kB Axis. J Agric Food Chem. (2023) 71:11929–40. doi: 10.1021/acs.jafc.3c02429, PMID: 37526282

[106] Liang H Sathavarodom N Colmenares C Gelfond J Espinoza SE Ganapathy V . Effect of acute TLR4 inhibition on insulin resistance in humans. J Clin Invest. (2022) 132:e162291. doi: 10.1172/JCI162291, PMID: 36066991 PMC9621129

[107] Mousa WK Al Ali A . The gut microbiome advances precision medicine and diagnostics for inflammatory bowel diseases. Int J Mol Sci. (2024) 25:11259. doi: 10.3390/ijms252011259, PMID: 39457040 PMC11508888

[108] Mocanu V Zhang Z Deehan EC Kao DH Hotte N Karmali S . Fecal microbial transplantation and fiber supplementation in patients with severe obesity and metabolic syndrome: a randomized double-blind, placebo-controlled phase 2 trial. Nat Med. (2021) 27:1272–9. doi: 10.1038/s41591-021-01399-2, PMID: 34226737

